# World's super scientists

**Published:** 2009

**Authors:** S. K. Jindal

The book written by the veteran chest physician Dr. O.A. Sharma is a collection of salient biographic features of 78 eminent scientists. The book certainly makes an interesting reading and provides some teasing and provocative information on the lives of few eminent people. The list includes the genius of the prebiblical era to that of the twentienth century – a large number belonging to the last three centuries.

It is rather a sad story of our busy lives that very few of us find time or incentive to study the different perspectives of medicine. Most of us remain content and overengaged in our daily clinical practice and in the study of text books or journals. Nonetheless, the history, including the life stores of scientists, is as or perhaps more important than the actual science. Each discovery has generally a long and hidden background history which frequently is more surprising and marvelous than the discovery itself. The journey is far more educative than the outcome. Similarly, the discoverer has a long tale of frustration and failures before tasting the success of his/her work. There is a lot more to know and learn from history than from the text books.

Of particular interest for the physicians are the stories of medical scientists and philosophers. The history dates back to as far as 5^th^ to 7^th^ centuries BC to Susruta and Hippocrates, Euclid, Aristotle, and Charaka. Unfortunately, the known and authentic information on these legends in the book is relatively meager. But we are told of several important facts about the lives of scientists of the medieval era and of the later period. The readers of ‘Lung India’ will enjoy to know more about Auenbrugger – the discoverer of percussion, whose father was a prosperous inn-keeper who used to confirm the level of wines in a barrel with the aid of percussion, and about Rene Laennec – the discover of auscultation, who for the first time used a thick paper rolled into a cylinder to listen to the heart sounds of a young woman since putting the ear on to her chest wall would have been embarrassing. Leonard Da Vinci was a multifaceted man as a painter, sculpturer, engineering designer, mathematician, and an anatomist – he had given detailed description of the heart chambers and its valves.

The book contains interesting information about other people with whose names we are generally familiar in respiratory medicine, such as Florence Nightingale or the ‘Lady with the Lamp’, Amedeo Avogadro (of the Avogadro's number fame), Andre Vesalius (the anatomist), Leeuwenhoek (the microscope man), John Dalton, Robert Boyle, Edward Jenner, Robert Koch, Paul Ehrlich, Wilhelm Roentgen, Louis Pasteur, and several others. Some of the important Indian inclusions, besides Susruta and Charaka are those of Aryabhatta, C.V. Raman, J.C. Bose, S. Ramanujam, and A.P.J. Abdul Kalam.

The author's choice is quite varied and personal. The author provides humor, a bit puzzling tail-piece, or an acrostic at the end of each chapter. It is neither a scholarly study nor an indepth story of the lives of these people. Most of the citations are generally casual. But the book may serve as an ‘appetizer’ before one goes for a full meal. It is a good part time while traveling or relaxing on a holiday.

